# Molecular functions of ANKLE2 and its implications in human disease

**DOI:** 10.1242/dmm.050554

**Published:** 2024-05-01

**Authors:** Adam T. Fishburn, Cole J. Florio, Nick J. Lopez, Nichole L. Link, Priya S. Shah

**Affiliations:** ^1^ Department of Microbiology and Molecular Genetics, University of California, One Shields Avenue, Davis, CA 95616, USA; ^2^ Department of Neurobiology, University of Utah, 20 South 2030 East, Salt Lake City, UT 84112, USA; ^3^ Department of Chemical Engineering, University of California, One Shields Avenue, Davis, CA 95616, USA

**Keywords:** Cell division, Microcephaly, Neurodevelopment

## Abstract

Ankyrin repeat and LEM domain-containing 2 (ANKLE2) is a scaffolding protein with established roles in cell division and development, the dysfunction of which is increasingly implicated in human disease. ANKLE2 regulates nuclear envelope disassembly at the onset of mitosis and its reassembly after chromosome segregation. ANKLE2 dysfunction is associated with abnormal nuclear morphology and cell division. It regulates the nuclear envelope by mediating protein-protein interactions with barrier to autointegration factor (BANF1; also known as BAF) and with the kinase and phosphatase that modulate the phosphorylation state of BAF. In brain development, ANKLE2 is crucial for proper asymmetric division of neural progenitor cells. In humans, pathogenic loss-of-function mutations in *ANKLE2* are associated with primary congenital microcephaly, a condition in which the brain is not properly developed at birth. ANKLE2 is also linked to other disease pathologies, including congenital Zika syndrome, cancer and tauopathy. Here, we review the molecular roles of ANKLE2 and the recent literature on human diseases caused by its dysfunction.

## Teaser

**Summary:** The scaffolding protein ANKLE2 is conserved throughout animals, with important roles in cell division and brain development. Disruption of ANKLE2 function is associated with several disease states.

## Introduction

ANKLE2 (also known as LEM4, KIAA0692 or MCPH16) is named after its LEM and ankyrin-repeat domains (Lee and Wilson, 2004). LEM domains form globular motifs composed of two parallel α-helices of ∼40 amino acid (aa) residues and are mostly found in inner nuclear membrane (INM) proteins (Cai et al., 2001) (Box 1). Named after the proteins in which they were first discovered – i.e. LAP2, emerin and MAN1 (Lin et al., 2000; Laguri et al., 2001), LEM domains mediate protein-protein interactions with barrier-to-autointegration factor (BANF1; also known and hereafter referred to as BAF) (Shumaker et al., 2001; Mansharamani and Wilson, 2005; Cai et al., 2007). BAF has distinct functions in the nucleus during different phases of the cell cycle. During interphase BAF has high mobility within the nucleoplasm where it binds chromatin and many nuclear proteins (Sears and Roux, 2020). During nuclear envelope reassembly BAF binds to chromatin and membrane-bound LEM-domain proteins, including ANKLE2, tethering DNA to the reforming nuclear envelope (Umland et al., 2000; Bradley et al., 2005; Samson et al., 2018) (Box 1). This creates a meshwork with the nuclear lamina to provide stability to the nuclear envelope.

**Box 1. Glossary**

**Affinity purification–mass spectrometry (AP–MS) analysis:** C-terminal Strep-tag II affinity tags are added to proteins of interest and expressed in appropriate cells (e.g. HEK293 T cells) by transfection. Cell lysate is harvested and applied to Strep-tag II-binding beads, which purifies the proteins of interest and any physically interacting proteins. The beads are washed, and bound proteins are eluted, processed, and submitted for mass-spectrometry analysis, which identifies and quantifies the proteins in the sample.

**Compound heterozygous:** Different mutations in a single gene occurring on separate chromosomes, which are inherited from each parent.

**cGAS–STING-mediated apoptosis:** Cytosolic DNA is sensed by cyclic GMP-AMP synthase (cGAS), resulting in transcriptional expression of cGAS–STING-induced interferons, which enhance apoptosis progression (Xu et al., 2023).

**DN4 thymocyte:** In the initial stages of T cell development within the thymus, precursor cells do not express CD4 and CD8, and are denoted as double-negative (DN) thymocytes. There are four early differentiation stages (DN1-4). DN4 thymocytes are the last stage of development before functional maturation is completed.

**Forward mosaic genetic screen:** Model organisms, such as *Drosophila*, or cell lines are mutagenized to generate stocks harboring random mutations. These stocks are then screened for various phenotypes. Samples with interesting phenotypes are sequenced to identify the mutated gene underlying the phenotype.

**Glutathione S-transferase (GST) pulldown:** A common biochemical technique to determine physical protein–protein interactions. A GST-fusion protein is expressed as bait, binding to a glutathione sepharose matrix. Cell lysate is incubated on the matrix, and proteins that interact with the bait are retained. These proteins are later eluted and can be detected using other methods.

**Guillan–Barré syndrome (GBS):** A rare autoimmune disorder in which the host immune system damages the myelin sheathes of peripheral nerves. Common symptoms are weakness in the extremities that, in severe cases, can cause paralysis or difficulty breathing.

**Inner nuclear membrane (INM):** The inner membrane of the double phospholipid nuclear envelope. It is rich of proteins involved in maintaining nuclear structure and chromatin organization.

**Intrinsically disordered protein:** Proteins or parts of a protein, which lack fixed, organized or stable three-dimensional structure. Disordered regions can serve as flexible linkers between other structured regions or act as linear motifs that can mediate interactions between the protein and other substrates (other protein, RNA, DNA, etc.) (Trivedi and Nagarajaram, 2022).

**Nuclear envelope:** The nucleus is surrounded by a double phospholipid membrane that separates the nucleoplasm from the rest of the cell. This barrier can be passed through nuclear pore complexes. The nuclear lamina on the inner side of the envelope is composed of filament lamin proteins and provides structure to the nucleus.

**Par complex:** A protein complex composed of proteins responsible for asymmetrically partitioning developmental determinants, allowing for variable daughter-cell-fate outcomes during embryogenesis.

**Random mutagenesis suppressor screen:** Model organisms – such as *C. elegans*, or cell lines that already harbor a genetic mutation causing a defined phenotype – are randomly mutagenized. These mutagenized populations are then screened for those that randomly acquire mutations that reverse the phenotype caused by the original genetic mutation.

**Telencephalon:** During vertebrate brain development, the brain initially forms three distinct sections, the forebrain (prosencephalon), midbrain (mesencephalon) and hindbrain (rhombencephalon). Later, the forebrain divides, developing into two parts, i.e. the telencephalon and the diencephalon. The telencephalon (also known as the cerebrum), is the largest part of the brain, containing multiple lobes with many functions each.

The second functional domain of ANKLE2 is its ankyrin-repeat domain. These domains are widely found in proteins across the tree of life. Composed of 33 aa residues with a clear consensus sequence (Kohl et al., 2003; Mosavi et al., 2004), these domains confer protein stability and scaffolding function for protein-protein interactions (Kumar and Balbach, 2021). They often occur in 2-7 tandem repeats, but can repeat up to 33 times (Mosavi et al., 2004). Given the high prevalence of ankyrin repeats in many proteins, their function is linked to many cellular processes, including molecule transport, adhesion, signaling, cytoskeletal stability and cell division. Unsurprisingly, mutations in the ankyrin repeats of these proteins contribute to a wide array of human diseases (Sharma et al., 2020).

The combination of both a LEM and ankyrin-repeat domain within a single protein is rare, and is shared only by ANKLE2 and ANKLE1 (also known as LEM3). Like ANKLE2, ANKLE1 interacts with chromatin via a physical interaction with BAF (Brachner et al., 2012). However, the functional similarities between these two proteins appear to end here, as ANKLE1 then acts as an endonuclease to cleave genomic or mitochondrial DNA (Brachner et al., 2012; Song et al., 2020; Przanowski et al., 2023). By contrast, ANKLE2 lacks any known enzymatic domains or activity and, instead, functions as a scaffold for other protein-protein interactions. Several canonical ANKLE2-protein interactions mediate the stability of the nuclear envelope, thereby assisting in cell division. However, proteomic studies suggest that ANKLE2 has many other interacting partners (Gupta et al., 2015; Hein et al., 2015; Go et al., 2021; Huttlin et al., 2021) and, as we discuss later, is speculated to have diverse roles in cell biology and human disease.

This Review aims to provide background on the molecular structure and function of ANKLE2 and relate this to human disease. We review reported insights into the molecular and cellular functions of ANKLE2 and, finally, explore recent findings that suggest novel roles for ANKLE2 in neurodevelopment, cancer, neurodegenerative diseases, immune system development and virus pathogenesis.

## Conservation of ANKLE2 molecular architecture

**Fig. 1.**
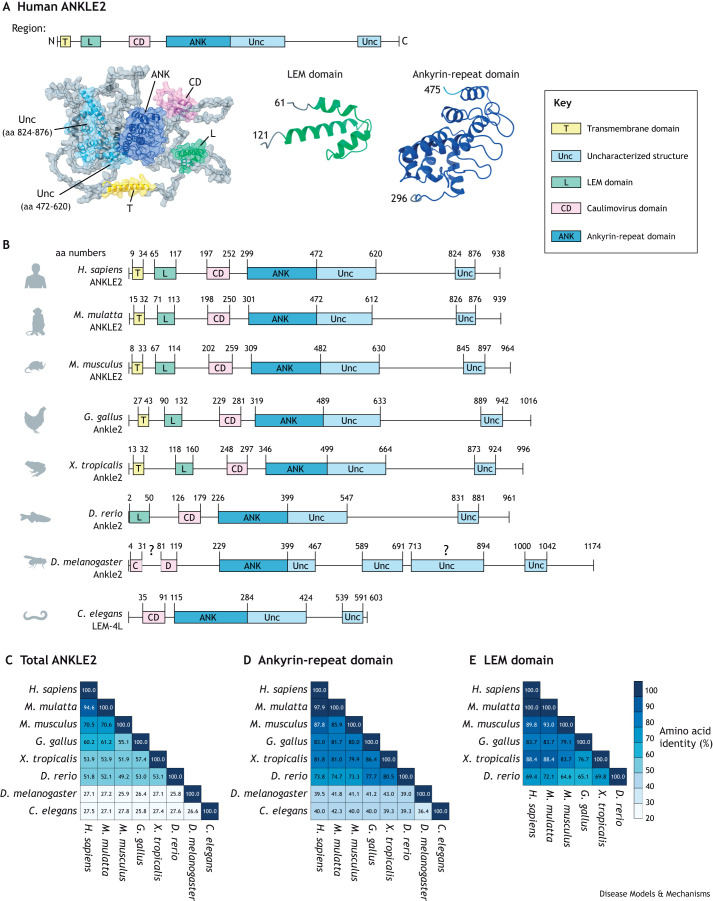
**Conservation of ANKLE2 structural domains.** (A) Prediction of human ANKLE2 (UniProt Q86XL3) protein structure by using the AlphaFold database, with annotated and uncharacterized (Unc) structural regions highlighted. C, C-terminus; N, N-terminus. (B) Protein structures of ANKLE2 and its orthologs in different species according to the National Center for Biotechnology Information (NCBI). Structured domains not previously annotated (such as LEM or ankyrin-repeat domain) were identified by using AlphaFold (Jumper et al., 2021; Varadi et al., 2022). Regions were considered structured when the AlphaFold per-residue estimate of its confidence (pLDDT) was >70 (on a scale from 0-100) for each given amino acid (aa) residue. Transmembrane domains were annotated using DeepTMHMM (https://dtu.biolib.com/DeepTMHMM) (Hallgren et al., 2022 preprint). The Caulimovirus domain in *D. melanogaster* Ankle2 appears to maintain a similar structure but is broken into two segments with a β-sheet-disordered region–α-helix-β sheet-α-helix organization. The region in *D. melanogaster* Ankle2 between aa residues 713-894 represents an uncharacterized structured region between region 5 and 6 without a defined orthologous region among other evaluated orthologs. (C-E) Conservation of aa residues among ANKLE2 orthologs as well as for specific protein domains (as shown in B) determined using Clustal Omega multiple sequence alignment (Sievers et al., 2011). Conservation of aa residues in the entire ANKLE2 protein sequence (C), the ankyrin-repeat domain (D) and the LEM domain (E), showing that these vital domains have higher degrees of conservation.

ANKLE2 is conserved throughout metazoans. LEM proteins and the LEM domain are proposed to have coevolved with BAF to act as INM tethers (Brachner and Foisner, 2011). ANKLE2 contains both characterized and uncharacterized structural domains predicted by the protein structure database AlphaFold, and the organization of these motifs within ANKLE2 is consistent across orthologs (Fig. 1). In humans and many other vertebrates ANKLE2 begins with an N-terminal transmembrane (TM) domain that acts as an anchor to the INM and endoplasmic reticulum (ER) membrane. In human cells loss of this TM domain causes ANKLE2 to mislocalize to the cytoplasm (Elkhatib et al., 2017; Fishburn et al., 2022a preprint). Interestingly, invertebrates do not have this TM domain (Fig. 1B), and Ankle2 of *Drosophila melanogaster* maintains localization to the INM and ER through an unknown mechanism (Link et al., 2019). The LEM domain follows the TM domain, and its standard helix-turn-helix structure is maintained throughout vertebrates (Fig. 1). Invertebrates have no recognizable LEM domain, although a LEM domain-equivalent might have a different organization or structure. Given the absence of a conserved LEM domain in invertebrates, it is especially interesting that the uncharacterized structure that follows the LEM domain (aa residues 197-252 in human ANKLE2) is present in all species examined (Fig. 1B). This structure consists of 50-60 aa residues and is predicted to form a β sheet-α helix-β sheet-α helix (Fig. 1A). In certain databases this region is annotated as a Caulimovirus viroplasmin VI domain, which – in *Caulimoviruses –* mediates the formation of viral inclusion bodies and acts as a site of virus assembly (Wintermantel et al., 1993). Hereafter, we refer to this structured region as the Caulimovirus domain. It is worth noting that no study has determined whether this region arose via horizontal gene transfer from these viruses or through convergent evolution. The potential function of the Caulimovirus domain in mediating specific ANKLE2 interactions are discussed in more detail later.

Following the Caulimovirus domain is the ankyrin-repeat domain, which acts as the scaffolding domain for protein-protein interactions (Fig. 1A). The aa residues of the LEM and ankyrin-repeat domains are more highly conserved across orthologs compared to the rest of the protein (Fig. 1C-E). Immediately following the ankyrin-repeat domain is a large structured region (aa residues 472-620 in human ANKLE2) of unknown significance or function. This presence of this region and its overall organization is broadly conserved (Fig. 1B). Given its proximity to the ankyrin-repeat domain, this structured region might stabilize or expand its function. In vertebrates the remainder of ANKLE2 appears to be disordered, except for a small structured region that coils back to interact with the previously mentioned uncharacterized structure. The space between each of these structured regions seemingly consists of intrinsically disordered protein regions (Box 1), which are known to regulate protein-protein interactions, and serve as sites for protein regulation and signaling (van der Lee et al., 2014; Wright and Dyson, 2015). The molecular functions of these uncharacterized structured regions are largely unknown. However, they likely play key roles in ANKLE2 function as mutations that disrupt these regions are linked to human disease, as we discuss later in this Review.

## Molecular and cellular functions of ANKLE2

Studies over the last decade have given insight into the cellular functions of ANKLE2; however, much is still unclear. In this section we review the known functions of ANKLE2 in aspects of cell division, T cell development, and asymmetric division of neural progenitor cells.

### Cell division

A major feature of cell division is the assembly and disassembly of the nuclear envelope. During eukaryotic interphase the nuclear envelope exists as a double lipid bilayer membrane. During mitosis the nuclear envelope must disassemble to allow for chromosome condensation in prophase and for chromatid segregation into the two daughter cells (Hetzer, 2010). Thereafter, the nuclear envelope must quickly and efficiently reassemble to enclose the DNA again. The initial disassembly of the nuclear envelope is triggered by phosphorylation of many nuclear envelope proteins (Heald and McKeon, 1990; Macaulay et al., 1995). This includes the DNA-binding protein BAF, which is phosphorylated by the widely expressed serine/threonine kinase VRK1. Despite being small, BAF has many binding partners and functions in the cell, the latter including gene regulation, DNA damage responses and defense against DNA viruses (Sears and Roux, 2020). Phosphorylation of BAF reduces its DNA-binding affinity (Marcelot et al., 2021) and alters its localization (Nichols et al., 2006). During interphase non-phosphorylated BAF is located diffusely throughout the nucleoplasm, where it interacts with chromatin (Haraguchi et al., 2007). Early in mitosis BAF is phosphorylated and becomes evenly distributed throughout the cytoplasm. After metaphase BAF is dephosphorylated and localizes to the DNA ‘core region’ around centromeres. Interestingly, BAF is the first protein to associate with this region, where it forms an immobile complex with other proteins to enable nuclear envelope reassembly to be initiated (Haraguchi et al., 2008). Depletion of BAF or of its kinase VRK1 dramatically alters nuclear envelope architecture, chromatin dynamics during mitosis and nuclear envelope reassembly in the roundworm *Caenorhabditis elegans* (Gorjánácz et al., 2007) as well as in human cell lines (Molitor and Traktman, 2014). Thus, the coordination of BAF phosphorylation and localization are of crucial importance for nuclear envelope dynamics, and for cell division in eukaryotic cells.

The first foundational study of ANKLE2 discovered its role in coordinating BAF phosphorylation and dephosphorylation in *C. elegans* (BAF-1 in *C. elegans*) and HeLa cells (Asencio et al., 2012). The *C. elegans* ortholog of human *ANKLE2* – named *lem-4 like* (*lem-4L*) due to the lack of a clear LEM domain – also plays a role in nuclear envelope formation and BAF regulation. Temperature-sensitive mutations in *lem-4L* are lethal in early *C. elegans* development and give rise to cells with defective nuclear morphology. Interestingly, a random mutagenesis suppressor screen (Box 1) yielded a *lem-4L C. elegans* mutant line that can grow at previously lethal temperatures because of a newly introduced P69L mutation in the *VRK1* ortholog (*vrk-1*). The use of RNA interference (RNAi) to silence *vrk-1* is tolerated in *lem-4L* mutants, despite being lethal in *lem-4L* wild-type embryos. Depletion *of vrk-1* in *lem-4L* mutants restores nuclear morphology*.* This suggests that mutation or reduction of *vrk-1* suppresses the aberrant effects of the *lem-4L* mutation. An analysis of BAF-1 phosphorylation states revealed that the silencing of *lem-4L*, but not other LEM genes (i.e. *lem-3, lem-2, emr-1*), increases BAF-1 phosphorylation. Whereas, the silencing or mutation of *vrk-1* dramatically decreases BAF-1 phosphorylation, consistent with its ability to rescue *lem-4L* mutant lethality. Interestingly, the silencing and/or mutation of both *lem-4L* and *vrk-1* leads to balanced phosphorylation of BAF-1. These opposing effects on BAF-1 phosphorylation indicate that LEM-4L plays a role in reversing BAF-1 phosphorylation by VRK-1. Glutathione-S-transferase (GST) pulldown experiments (Box 1) revealed that LEM-4L and VRK-1 physically interact, as do their human orthologs (ANKLE2 and VRK1), although it is unclear if this physical interaction is limited to a particular phase of the cell cycle. *In vitro* kinase assays revealed that both LEM-4L and human ANKLE2 can inhibit the VRK-1-mediated phosphorylation of BAF-1 in a concentration-dependent manner, via a mechanism that, initially, had been unclear (Asencio et al., 2012).

ANKLE2 also interacts with phosphatases. Proteomics studies have previously reported that human ANKLE2 interacts with several subunits of the serine/threonine protein phosphatase 2A (PP2A) complex (Bollen et al., 2009; Glatter et al., 2009). This complex has a broad substrate range and controls cell cycle entry and exit (Bollen et al., 2009; Hunt, 2013; Wlodarchak and Xing, 2016). Asencio et al. demonstrated in their 2012 study that ANKLE2 and PP2A (specifically subunits PP2A-C, PP2A-R1, and PP2A-B55α) physically interact, and that PP2A can directly dephosphorylate BAF *in vitro* (Asencio et al., 2012). Interestingly, a truncated portion of ANKLE2 (aa residues 162-349) can interact with all these PP2A subunits, but a smaller truncated form (aa residues 255-349) that excludes the Caulimovirus domain (aa residues 197-252) cannot. This suggests that the Caulimovirus domain supports the interaction with PP2A. Depletion of these PP2A subunits in HeLa cells by using RNA interference (RNAi) decreases the anaphase recruitment of BAF to chromatin, indicating that PP2A regulates the phosphorylation state of BAF to control mitosis. These trends were mirrored in *C. elegans* upon the silencing of *let-92* and *tag-93,* the orthologs of human PP2A-C and PP2C-B, respectively (Asencio et al., 2012).

Further studies in human cells expanded our knowledge of the various cellular functions of ANKLE2. Snyers et al. used ANKLE2-deficient HeLa cells to determine which regions of ANKLE2 mediate BAF-chromatin association during cell division (Snyers et al., 2018). *ANKLE2* knockout HeLa cells expressing GFP-tagged BAF were analyzed using fluorescence microscopy. In these ANKLE2-deficient cells, association of BAF with chromatin was dramatically reduced and BAF was diffusely located in the cytoplasm. To determine which regions of ANKLE2 mediate this process, the authors generated various ANKLE2 truncation mutations and introduced them into the *ANKLE2* knockout cells. Surprisingly, in mutants lacking the TM or LEM domain, association of BAF with chromatin was restored. However, three mutants that lacked the Caulimovirus domain or C-terminal uncharacterized region of ANKLE2 (ANKLE2 241-938, 311-938, or 1-822) failed to restore this association (Snyers et al., 2018). This further highlights the importance of the Caulimovirus domain and suggests that these conserved regions, for which the molecular functions are not fully understood, play vital roles in how ANKLE2 regulates BAF (Fig. 1).

Another study has highlighted a potential mechanism to regulate how ANKLE2 controls nuclear envelope reassembly. In 2016, Kaufmann et al. reported that ANKLE2 and the deacetylase sirtuin 2 (SIRT2) physically interact *in vitro* (Kaufmann et al., 2016). SIRT2 has many substrate proteins that collectively regulate microtubule dynamics and cell cycle progression (Inoue et al., 2007). Both depletion and overexpression of SIRT2 lead to abnormal nuclear morphologies in U2OS cells, mimicking cells with depleted ANKLE2 (Kaufmann et al., 2016). However, overexpression of ANKLE2 does not impact nuclear morphology. Kaufmann et al. then showed that SIRT2 directly deacetylates ANKLE2, and regulates the acetylation and phosphorylation of ANKLE2 during the cell cycle. Importantly, they also found that acetylation of aa residue K302 in the N-terminal part of the ankyrin-repeat domain of ANKLE2 is important for nuclear envelope reassembly in U2OS cells (Kaufmann et al., 2016).

**Fig. 2.**
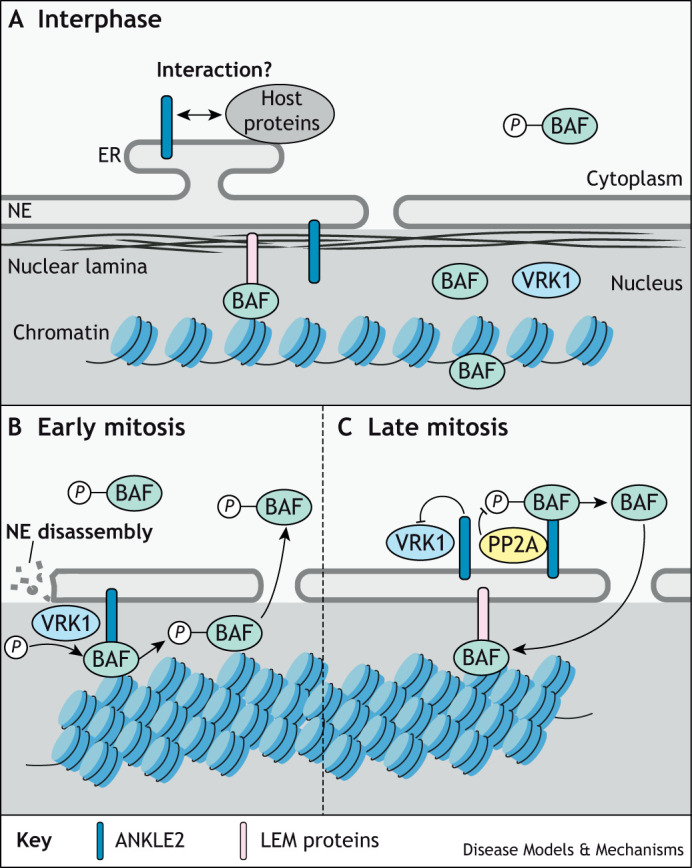
**Role of ANKLE2 in nuclear envelope disassembly and reassembly via its regulation of BAF phosphorylation.** (A) During interphase, LEM-domain proteins contribute to chromatin organization by acting as a bridge to DNA, BAF, nuclear lamina and the inner nuclear membrane. Non-phosphorylated BAF is diffusely spread throughout the nucleoplasm and may interact with other proteins, while some phosphorylated BAF exists in the cytoplasm. ANKLE2 is localized to the inner nuclear membrane and to the ER where it is thought to interact with other host proteins and may have other roles in the cell. (B) During early mitosis, ANKLE2 facilitates the phosphorylation of BAF through VRK1. Phosphorylated BAF loses its affinity for DNA and relocalizes to the cytoplasm, allowing nuclear envelope (NE) disassembly and chromosome condensation. (C) During late mitosis, ANKLE2 inhibits VRK1 phosphorylation of BAF and facilitates BAF dephosphorylation through the PP2A-complex. This reinstates BAF DNA-binding, leading to nuclear envelope reassembly by recruitment of membrane-bound LEM proteins.

Together, these experiments established the model of ANKLE2-mediated control of nuclear envelope disassembly and reassembly. During interphase non-phosphorylated BAF is present in the nucleoplasm, where it may bind to chromatin and the nuclear envelope via lamins and LEM proteins, including ANKLE2. During interphase some fraction of phosphorylated BAF may exist in the cytoplasm (Haraguchi et al., 2007; Berk and Wilson, 2016). On mitotic entry VRK1 phosphorylates the remaining nuclear BAF, reducing its affinity for DNA and its nuclear retention. Later ANKLE2 inhibits the VRK1-mediated phosphorylation of BAF and PP2A dephosphorylates BAF, presumably via its physical interactions with ANKLE2. This restores the binding of DNA to BAF, allowing BAF to concentrate at the ‘core region’ after anaphase and to initiate nuclear envelope reassembly (Fig. 2).

*ANKLE2* is expressed throughout the body in human adults (Fagerberg et al., 2014) and likely plays a fundamental role in mitosis. In humans, *ANKLE2* is seemingly expressed throughout development, from 10 weeks post conception. Its expression has also been observed in all seven evaluated tissues (brain, cerebellum, heart, kidney, liver, ovary and testis), supporting that *ANKLE2* is ubiquitously expressed in humans throughout life (Cardoso-Moreira et al., 2019). It is also expressed throughout development in other organisms, including mice (*Mus musculus*) (Yue et al., 2014), zebrafish (*Danio rerio*) (White et al., 2017) and fruit flies (*D. melanogaster*) (Brown et al., 2014). In the remainder of this section, we review what we know about the temporospatial patterns of *ANKLE2* expression and what this tells us about its specific roles in development.

### Immune cell development

ANKLE2 has been recently implicated in immune cell development, and specifically in T cell maturation. This occurs through transcriptional regulation of *ANKLE2* by the zinc finger protein 335 (ZFP335), which regulates *ANKLE2* expression by binding to its promoter. ZFP335 is a C2H2 zinc-finger transcription factor that is essential for vertebrate embryonic development and functions in T cell maturation (Yang et al., 2012; Han et al., 2014). In mice that harbor the R1092W point mutation of Zfp335, binding of Zfp335 to the *Ankle2* promoter is abolished, reducing expression of *Ankle2*. This loss of Ankle2 is accompanied by T cell maturation defects throughout multiple stages of T cell development and results in decreased number of T cells. *In vitro* overexpression of mouse *Ankle2* in a Zfp335 mutant background partially rescues T cell maturation. This finding indicates that Ankle2 is a downstream target of Zfp335 and required for T cell maturation (Han et al., 2014). Another recent study found that the continuous expression of Zfp335 and Ankle2 is necessary for DN4 thymocyte (Box 1) survival in mice, and for the proper development of late-stage T cells in the thymus. Han et el. hypothesized that decreased levels of Ankle2 lead to increased levels of hyperphosphorylated Baf, and, thus, disrupt nuclear envelope assembly. The resulting nuclear envelope morphology defects would, in turn, lead to increased levels of cytoplasmic DNA, thereby activating cGAS–STING-mediated apoptosis (Box 1). Thus, the loss of Zfp335 resulted in impaired T cell development due to the disruption of the Zfp335/Ankle2/Baf axis, ultimately triggering DN4 cell death through cGAS–STING signaling (Ratiu et al., 2022). These findings are supported by previous studies showing that depleted levels of ANKLE2 (and of its orthologs) disrupt nuclear envelope morphology (Asencio et al., 2012; Link et al., 2019; Apridita Sebastian et al., 2022). In human cells, BAF has direct roles in cGAS–STING signaling as a modulator of basal cell-intrinsic immunity in response to viral infection. It does so by regulating cGAS-dependent interferon-stimulated gene homeostasis, as well as affecting the levels of cytoplasmic DNA (Ma et al., 2020). However, whether ANKLE2 directly participates in this signaling is unknown.

### Asymmetric cell division in neural progenitor cells

**Fig. 3.**
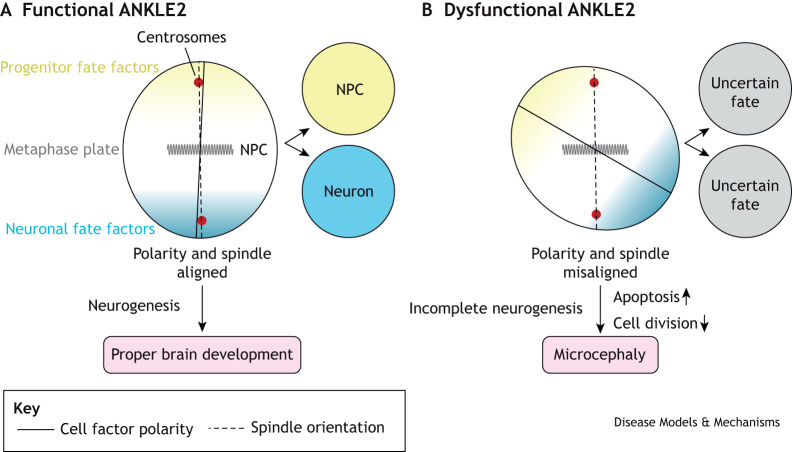
**Model of ANKLE2-mediated dysregulation of asymmetric division in neural progenitor cells, leading to microcephaly.** (A) During brain development, neural progenitor cells (NPCs) divide asymmetrically, giving rise to two daughter cells: one cell acquires a neuronal fate (neuron) and the other is retained as a NPC. These different daughter cell fates are brought about by the polarization of fate-determining factors in NPCs across the metaphase plate. Thus, this process depends on the alignment of cell polarity with the mitotic spindle and is required for proper neurogenesis and brain development. (B) In ANKLE2-deficient cells the polarity of cell fate factors becomes misaligned relative to the mitotic spindle. The resulting dysregulation of NPC polarity leads to a reduction in neuronal cell numbers generated during neurogenesis, an effect that is compounded by increased apoptosis and decreased cell division, resulting in microcephaly.

ANKLE2 also plays a specialized role in regulating asymmetric cell division in neural progenitor cells (NPCs). To maintain stemness, NPCs segregate their stem-like proteins asymmetrically during cell division. The mutation of *Ankle2* in *D. melanogaster* results in aberrant NPC division, with reduced cell proliferation and increased cell death (Yamamoto et al., 2014). The depletion or mutation of *D. melanogaster Ankle2* also leads to the disruption of the ER and nuclear envelope, which in turn causes the release of the VRK1 ortholog Ball, into the cytosol. Interestingly, this is accompanied by defects in NPC polarity and spindle alignment, which are both required for asymmetric cell division. There are also defects in the phosphorylation of atypical protein kinase C (aPKC) in *D. melanogaster Ankle2* mutants. aPKC is part of the Par complex, together with Par-3 and Par-6. During mitosis this complex is apically located in NPCs, and the activity of this complex ensures asymmetric cell division proceeds properly. Phosphorylation of aPKC is thought to be associated with its activation (Kim et al., 2009) (Box 1). The Par complex is negatively regulated by physical interaction between aPKC and Lethal (2) giant larvae [L(2)gl], but when aPKC is active it phosphorylates L(2)gl to prevent it from binding aPKC (Betschinger et al., 2003; Rolls et al., 2003). Defects in NPC asymmetric division in *Ankle2* mutant *D. melanogaster* can be rescued by partial loss of *ball* or a temperature-sensitive mutation in *l(2)gl*, suggesting both Ball and L(2)gl are overactive in *Ankle2* mutants, and providing a genetic link between Ankle2 and the Par complex (Link et al., 2019) (Fig. 3).

In summary, ANKLE2 has multiple roles in cell biology through its functions in regulating nuclear envelope dynamics, T cell maturation and asymmetric cell division. Next, we expand our review to the physiological human diseases caused by disruption of ANKLE2 function.

## Roles of ANKLE2 in human disease

The control of cell division during development is a complex and carefully coordinated process that can be perturbed in many ways – with often disastrous consequences for a cell and/or organism. Given the role of ANKLE2 in regulating cell division, its altered or inhibited function can lead to severe cellular defects that, ultimately, result in dysregulated neurodevelopment or cancer.

### Microcephaly

Microcephaly describes a neurological condition in which brain and head size are greatly reduced. In clinical terms, someone with a head circumference of 2–3 standard deviations (SD) below the mean for their age and sex is classified as having microcephaly, with a reduction of >3 SD being classified as severe microcephaly (Jayaraman et al., 2018). While head circumference is the main criterium for diagnosing microcephaly, it is often characterized by a disproportionally small brain and head size relative to the rest of the face and body.

Microcephaly can be caused by a number of factors, including toxins (Feldman et al., 2012); pathogen infection during pregnancy (Giles et al., 1965; McLeod et al., 2006; Mlakar et al., 2016; Messinger et al., 2020); metabolic conditions, such as maternal phenylketonuria (Waisbren et al., 2015); traumatic brain injuries (Lo et al., 2003) and genetic mutations in a wide array of genes (Alcantara and O'Driscoll, 2014; Naveed et al., 2018; Shaheen et al., 2019). Microcephaly can be further broken down into two main types, primary and secondary microcephaly. Primary microcephaly, i.e. microcephaly primary hereditary (MCPH), describes microcephaly that is present at birth and is usually due to neurodevelopmental defects. Secondary microcephaly occurs when a brain that has a normal size at birth does not grow appropriately with age. There is no cure for microcephaly, nor are treatments available to restore brain size or growth. The condition is commonly accompanied by seizures, severe developmental delays and impaired motor, vision or auditory functions (Jayaraman et al., 2018).

**Fig. 4.**
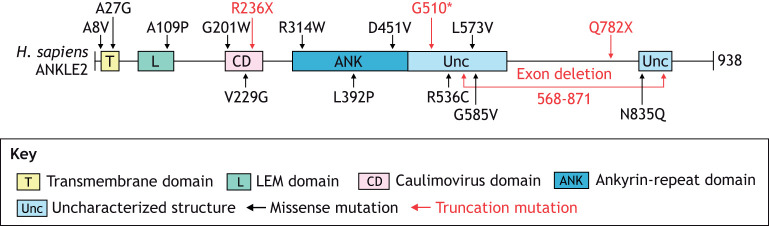
**Pathogenic mutations in human ANKLE2 associated with primary congenital microcephaly.** Schematic of human ANKLE2 protein structure showing sites of known pathogenic mutations, as originally described (Yamamoto et al., 2014; Link et al., 2019; Shaheen et al., 2019; Masih et al., 2022; Thomas et al., 2022). Point mutations leading to missense mutations are shown in black, mutations leading to protein truncations are shown in red. G510* indicates a hypothetical splicing mutation that leads to a premature protein termination (c.1421-1G>C), as identified in a compound heterozygous individual together with the A109P point mutation (Link et al., 2019). Allele combinations are listed in Table 1.

Genetic MCPH was first identified through autozygosity mapping in two consanguineous families, which revealed a genetic locus (*MCPH1*) for autosomal recessive MCPH (Jackson et al., 1998). Currently, there are 30 known genes whose mutations cause primary microcephaly, and more MCPH genes are identified almost every year, suggesting that yet more genes contribute to this condition (Carvalhal et al., 2022). Of the 30 genes identified so far, more than 20 are linked to the molecular regulation of mitosis (Amberger et al., 2019; Degrassi et al., 2020; Carvalhal et al., 2022). ANKLE2 was first discovered to cause MCPH in *D. melanogaster* after using a forward mosaic genetic screen (Box 1), which identified a neurodevelopmental phenotype in an *Ankle2* mutant (Yamamoto et al., 2014). At the third-instar larval stage of development flies harboring the L326H point mutation in *Ankle2* showed a reduced brain size without an overall growth defect (Yamamoto et al., 2014; Link et al., 2019). In CRISPR-generated *Ankle2* null mutant flies brain size was even further decreased, and associated with smaller overall size of the animal as well as failure to survive beyond the third-instar stage (Link et al., 2019). A corresponding search of whole-exome sequencing data identified a patient who recessively inherited MCPH through a compound heterozygous *ANKLE2* mutation. This compound mutation consisted of one allele with an L573V missense mutation and another allele with the nonsense mutation Q782X. Leucine residue L573 lies in the structured region following the ankyrin-repeat domain and the Q782X point mutation results in truncated ANKLE2 lacking the last structured region (Figs 1 and 4). This individual presented with severe microcephaly at birth and, at age 5.5 years, had a frontal-occipital circumference of −9 SD. A sibling with the same mutations also had severe microcephaly but, unfortunately, died shortly after birth. To establish whether human ANKLE2 is directly responsible for the neurodevelopmental defects diagnosed in this family, a rescue experiment was performed by expressing human ANKLE2 in *Ankle2* mutant flies. Expression of wild-type human ANKLE2 restored brain development to near normal levels, highlighting the functionally conserved role of ANKLE2 in brain development between humans and *Drosophila* (Yamamoto et al., 2014; Link et al., 2019).

**Table 1.**
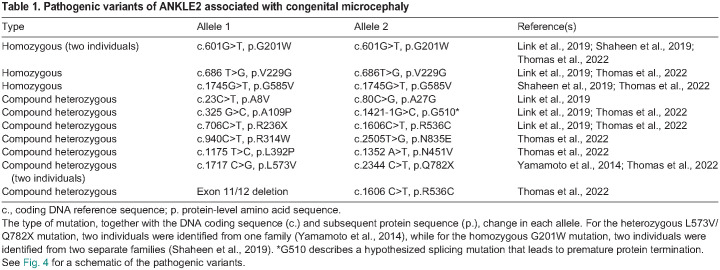
Pathogenic variants of ANKLE2 associated with congenital microcephaly

Due to its association with MCPH, *ANKLE2* is sometimes called MCPH16 – the clinical name for ‘microcephaly 16, primary, autosomal recessive’, the disorder caused by mutations in *ANKLE2.* Additional studies have expanded the range of *ANKLE2* mutations and allele combinations associated with this disease (Fig. 4 and Table 1) (Link et al., 2019; Masih et al., 2022; Thomas et al., 2022). Other studies have also implicated mutations of *VRK1* (Shaheen et al., 2019) and *ZFP335* (Yang et al., 2012) in MCPH, further supporting a role for the ANKLE2 pathway in neurodevelopment. In addition, in-depth imaging and clinical evaluation of ANKLE2-associated microcephaly in humans revealed twelve additional cases of MCPH16, and new pathogenic *ANKLE2* variants located in various domains of *ANKLE2* (Fig. 4 and Table 1). In ten of these cases, MCPH16 patients had missense mutations, and in two cases patients had nonsense mutations that resulted in premature protein termination. These pathogenic mutations were associated with a broad range of structural brain abnormalities and developmental delays, with speech and language delay being the most common abnormality (Thomas et al., 2022).

In addition to clinical studies, animal models have elucidated how ANKLE2 regulates brain development and how mutations in *ANKLE2* lead to the formation of MCPH16. While pathogenic mutations in *ANKLE2* were first uncovered in the invertebrate model *D. melanogaster* (Yamamoto et al., 2014; Link et al., 2019), the role of ANKLE2 in vertebrate brain development has only recently been explored using a zebrafish model (Apridita Sebastian et al., 2022). Surprisingly, in zebrafish, an *ankle2-*null mutation resulted in normal body and brain size at 6- or 14-days post fertilization (dpf). However, at 14 dpf, radial glial progenitor cell numbers and locomotor activity were significantly reduced. Reduced brain size in adult *ankle2* null mutant fish manifested later, at 3-4 months post fertilization (mpf), with the cerebellum and telencephalon (Box 1) being primarily impacted. These brain regions are consistent with those impacted in humans with pathogenic *ANKLE2* mutations, despite the delay in phenotype onset in zebrafish. While brain size is clearly affected in these animals, it is unclear if these impacts are specific or accompanied by a general reduction in animal size. Nonetheless, the ‘small brain’ phenotype could be rescued using morpholino-based *vrk1* knockdown, recapitulating the previously established relationship between these two genes (Asencio et al., 2012; Link et al., 2019). Finally, in this zebrafish model, the depletion of *ankle2* also led to infertility due to defective spermatogenesis that was partially rescued by mutation of *vrk1* (Apridita Sebastian et al., 2022)*.* The finding that *vrk1* is involved in fertility is supported by observations from *D. melanogaster*, in which *vrk1* mutations also lead to fertility defects (Cullen et al., 2005; Ivanovska et al., 2005; Lancaster et al., 2007). The impact of ANKLE2 on fertility and on spermatogenesis are yet to be explored in humans, although alternative *ANKLE2* transcripts have been identified in human spermatid cells (Elkhatib et al., 2017).

### Congenital Zika syndrome

Between 2015 and 2016 Zika virus (ZIKV) emerged as a global public health threat due to an epidemic across South and Central America. In healthy adults ZIKV is often clinically mild, except for rare cases of Guillan–Barré syndrome (Cao-Lormeau et al., 2016) (Box 1). In pregnant women ZIKV can be vertically transmitted and *in utero* infections can cause congenital Zika syndrome (CZS). CZS is characterized by a range of significant birth defects, including congenital contractures, ocular abnormalities (Campo et al., 2017), hip displacement (da Fonseca et al., 2023) and, in the most severe cases, MCPH (de Araújo et al., 2016; Mlakar et al., 2016; Moore et al., 2017). The occurrence of microcephaly after congenital ZIKV exposure is ∼5% and varies dramatically with gestational timing (Roth, 2022) as well as many other environment- and virus-associated factors (Adachi et al., 2020; Nunes et al., 2021). CZS-associated microcephaly has a mortality rate of ∼10%, but this also varies with severity of disease and other factors (N. Costa et al., 2020). Similar to other etiologies of microcephaly, CZS-associated microcephaly can present with multiple neurological defects, including ventriculomegaly, hypoplasia, simplified gyral patterns and calcifications (de Fatima Vasco Aragao et al., 2016). The molecular mechanisms by which ZIKV causes microcephaly are still not fully understood and are likely to be multifactorial (Fishburn et al., 2022b).

To investigate the molecular mechanisms of CZS, we assessed protein interactions between ZIKV and host proteins using a global proteomics approach (Shah et al., 2018). In these experiments, individual ZIKV proteins were expressed in HEK293T cells and subjected to affinity purification–mass spectrometry analysis (Box 1) to identify protein–protein interactions between each viral protein and the host proteome. This pipeline identified hundreds of high-confidence ZIKV-host protein–protein interactions, including an interaction between ZIKV non-structural protein 4A (NS4A) and host ANKLE2 (Shah et al., 2018). Transgenic expression of ZIKV NS4A under different ubiquitous or tissue-specific promoters in *D. melanogaster* larvae induced a ‘small brain’ phenotype (Shah et al., 2018). As in *Ankle2* mutant flies, this virally induced ‘small brain’ phenotype could be rescued by expression of human ANKLE2, by partial loss of the *VRK1* ortholog *ball* or by a temperature-sensitive mutation in the polarity regulator *l(2)gl* (Link et al., 2019)*.* However, expression of the pathogenic loss-of-function ANKLE2 point mutant Q782X failed to rescue this ‘small brain’ phenotype, further underlining inhibition of ANKLE2 by ZIKV NS4A (Yamamoto et al., 2014; Shah et al., 2018; Link et al., 2019). Interestingly, while *Ankle2* heterozygous animals had normal brain development, expression of ZIKV NS4A in these heterozygous animals led to more severe brain development phenotypes than observed for ZIKV NS4A expression in wild-type animals (Shah et al., 2018). This suggests that typically non-pathogenic variation in *ANKLE2* may sensitize individuals to ZIKV-induced microcephaly, tipping the scales from haplosufficiency to haploinsufficiency. This could also provide a host genetic basis for the spectrum of clinical outcomes observed in CZS.

**Fig. 5.**
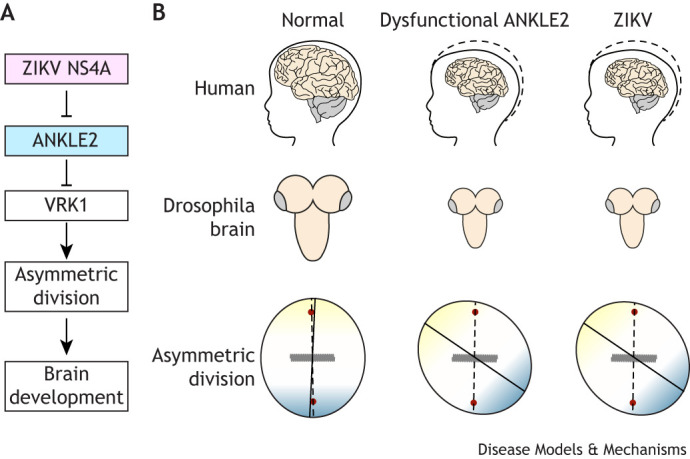
**Zika virus (ZIKV) NS4A inhibits ANKLE2 and causes similar pathogenic outcomes.** During mitosis, ANKLE2 interacts with BAF, VRK1 and PP2A to regulate nuclear envelope dynamics. During asymmetric cell division the ANKLE2-VRK1 pathway is crucial for establishing proper cell polarity. (A) Zika virus (ZIKV) NS4A interacts with ANKLE2 and inhibits its function to cause microcephaly. (B) Top row: Simplified schematic showing normal development of human brain (left) compared to microcephaly arising from dysfunction ANKLE2 (middle) or from congenital Zika syndrome (top). Middle and bottom rows: The mirrored ‘small brain’ phenotype in larval *Drosophila* brain (middle) and respective defects in asymmetric neuroblast division (bottom).

In addition to inhibiting gross brain development, ZIKV NS4A also inhibits cellular functions of Ankle2*.* In the same *Drosophila* model described above (Shah et al., 2018), expression of ZIKV NS4A induced disruption of NPC polarity and spindle alignment in wild-type flies similar to disruptions seen in *Ankle2* mutants (Link et al., 2019) (Fig. 5). Together, these findings suggest that ZIKV NS4A interacts with the host ANKLE2 to inhibit its functions in cell division, subsequently dysregulating NPC development and leading to microcephaly. Identifying the physical determinants of this protein–protein interaction is vital in understanding the mechanism how ZIKV NS4A inhibits ANKLE2. While these determinants are not fully established, they are of continuing interest to our group (Fishburn et al., 2022a preprint).

### Cancer

Function of ANKLE2 as a mitotic regulator also has implications in ovarian and breast cancer. Cancers arises because of defects in cell cycle control mechanisms leading to abnormal mitosis and unchecked cell proliferation. In high-grade serous ovarian carcinoma (HGS-OvC), *ANKLE2* is part of a network of 12 genes that interact with *VIRMA*, an RNA methylation/adenylation gene that contributes to tumor aggressiveness through N6-methylation of adenosine (m6A), ultimately targeting RNA as their ultimate destination (Miranda-Gonçalves et al., 2021). Using RNAi to silence *ANKLE2* in various human ovarian cancer cell lines (SKOV3, OVCAR, and APOCC) decreases cell viability and cell migration, and increases chemosensitivity to paclitaxel, a common chemotherapy used in ovarian cancer (Al-Farsi et al., 2022). Similarly, in estrogen receptor-positive (ESR^+^) human breast cancer cells lines (T47D, BT474 and MCF7), ANKLE2 overexpression contributes to tamoxifen resistance and accelerated tumor growth (Gao et al., 2018). In ESR^+^ human breast cancer cells ANKLE2 acts as a scaffold by stabilizing and facilitating the phosphorylation of estrogen receptor alpha (ESRα) through aurora-A kinase, thus activating ERα signaling, and increasing DNA binding and transactivation. Phosphorylated ERα directly targets cyclin D to cause tamoxifen resistance (Stendahl et al., 2004). Gao et al., 2018 also showed that ANKLE2 facilitates phosphorylation of the tumor suppressor retinoblastoma protein (Rb), activating the cyclin D–CDK4–Rb signaling axis to promote tamoxifen resistance by sending the cell from G1 to S phase (Gao et al., 2018). For other cancers, such as prostate adenocarcinoma, LEM-domain-containing proteins – including ANKLE1, EMD and LEMD2 – can serve as prognostic markers. However, in this type of prostate cancer, no significant changes in ANKLE2 expression were found (He et al., 2022).

## Conclusions

ANKLE2 is a multifunctional protein with established and emerging roles throughout the cell. In its most-studied function in cell division, ANKLE2 acts as a scaffold to regulate BAF phosphorylation and control nuclear envelope dynamics. These roles in cell division are directly linked to development in multicellular organisms. While clearly important for brain development and neurogenesis, the ubiquitous expression of ANKLE2 implies it has broader functionality. We speculate that ANKLE2 has at least two functionalities, depending on its subcellular localization and cell type. At the INM it interacts with and regulates BAF, while at the ER its interactions or roles are not understood. This hypothesis arises from the observation that ANKLE2 has distinct and conserved localization to the ER, while most LEM-domain-containing proteins are primarily nuclear or retained to the INM. This observation, along with the ubiquitous expression of ANKLE2, suggests the potential for a broader post-mitotic function in the ER. Our group has explored this unknown ER function and, recently, showed that it may be coopted for ZIKV replication (Fishburn et al., 2022a preprint). It is enticing to speculate that the scaffolding function of ANKLE2 could be hijacked by viral proteins, including ZIKV NS4A, to mediate aspects of virus replication in the ER that are otherwise inefficient.

Although not as well established, a post-mitotic function of ANKLE2 may also regulate the development of tauopathies. Tauopathies, including Alzheimer's disease, are a group of neurodegenerative disorders characterized by the aggregation of tau protein as intracellular neurofibrillary tangles within neurons (Wood et al., 1986). Speculation is that these aggregations are driven by changes in tau phosphorylation (Xia et al., 2020; Meng et al., 2022), and ANKLE2 may, in fact, prevent tau aggregation. Knockdown of *ANKLE2* in HEK293T tau biosensor cells led to the aggregation of insoluble and phosphorylated tau, as well as nuclear proteins, such as BAF, in the cytoplasm (Prissette et al., 2022). Another study involving *ANKLE2* knockdown in similar cells identified ANKLE2 as a key regulator in the development of both exosomal and vesicle-free aggregates of tau outside of the cell (Polanco et al., 2022). Together, these studies raise the potential for a post-mitotic role of ANKLE2 in regulating tau phosphorylation. While the mechanism by which ANKLE2 interacts with and regulates tau is still unclear, these data suggest a potentially important role for ANKLE2 in the development of neurodegenerative diseases. Notably, we recently showed that ZIKV NS4A not only inhibits fly brain development in an ANKLE2-dependent manner, but it also causes retinal neurodegeneration in adult flies (Link et al., 2024). It is interesting to speculate that this degenerative phenotype is also ANKLE2-dependent and disrupts a post-mitotic role of ANKLE2.

Future research is necessary and warranted to explore these possibilities and the roles of ANKLE2 in human diseases. As a scaffolding protein, evaluating protein-protein interactions is an obvious avenue to identify potential processes in which ANKLE2 is involved. A current significant challenge is the lack of an experimentally determined structure, complicated by many disordered regions. A well-defined structure would allow for a clearer understanding of how ANKLE2 interacts with other host proteins, such as BAF, PP2A, VRK1, tau or others. This might also illuminate how naturally occurring mutations or ZIKV NS4A may inhibit these interactions to cause disease. Understanding how this set of ANKLE2-interacting proteins varies between different stages of development (NPCs versus post-mitotic neurons) or cell states (interphase versus mitotic, healthy versus diseased, etc.) will provide new insights into the dynamic nature of ANKLE2 and how changes in these interactions can cause disease.
